# From Desert to Medicine: A Review of Camel Genomics and Therapeutic Products

**DOI:** 10.3389/fgene.2019.00017

**Published:** 2019-02-19

**Authors:** Amanat Ali, Bincy Baby, Ranjit Vijayan

**Affiliations:** Department of Biology, College of Science, United Arab Emirates University, Al Ain, United Arab Emirates

**Keywords:** camel genome, breeds, adaptations, immunogenomics, antibodies, anticancer

## Abstract

Camels have an important role in the lives of human beings, especially in arid regions, due to their multipurpose role and unique ability to adapt to harsh conditions. In spite of its enormous economic, cultural, and biological importance, the camel genome has not been widely studied. The size of camel genome is roughly 2.38 GB, containing over 20,000 genes. The unusual genetic makeup of the camel is the main reason behind its ability to survive under extreme environmental conditions. The camel genome harbors several unique variations which are being investigated for the treatment of several disorders. Various natural products from camels have also been tested and prescribed as adjunct therapy to control the progression of ailments. Interestingly, the camel employs unique immunological and molecular mechanisms against pathogenic agents and pathological conditions. Here, we broadly review camel classification, distribution and breed as well as recent progress in the determination of the camel genome, its size, genetic distribution, response to various physiological conditions, immunogenetics and the medicinal potential of camel gene products.

## Introduction

**FIGURE 1:**
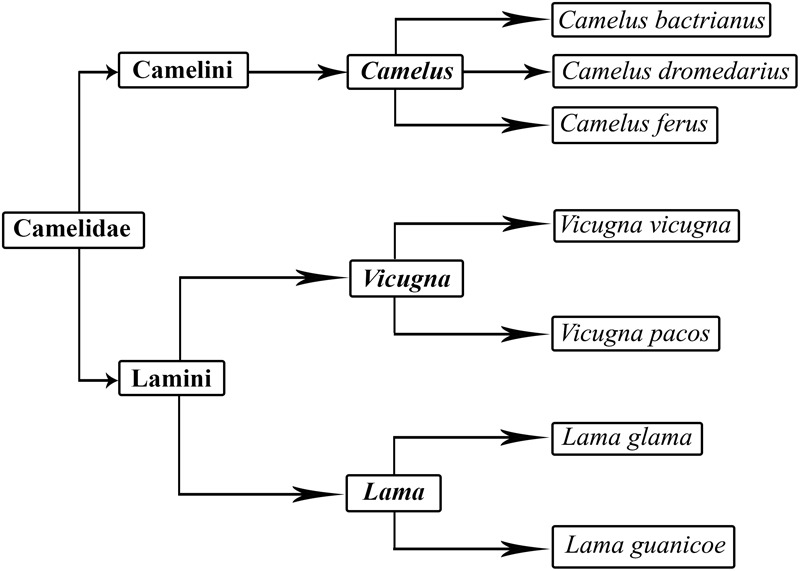
Classification of camels.

Camels contribute hugely to human survival in less agroecological parts of African, Asian and Arabian deserts. They have been used for transportation, as a source of food and for protection for a very long time. Nowadays, they are hugely important in many parts of the arid world as sustainable livestock species (Burger, 2016). Camels are members of the Camelidae family. Camelidae probably appeared in North America around 35 million years ago during the Eocene period (Reed, 1972). There are two major types – small and large camels – which are further subdivided into *Camelus, Lama*, and *Vicugna* genera. A clearly defined universal classification of camels does not exist. However, the most widely accepted classification is given in Figure 1 (Wu et al., 2014). They are generally differentiated on the basis of color, function and habitat. Camel breeds have roughly the same shape but diverge in body conformation, size and color (Al-Swailem et al., 2010). Large camelids include two domestic species: *Camelus dromedarius*, the single-humped camel, and *Camelus bactrianus*, the two-humped camel. The dromedary camels are also known as Arabian camels and are mostly prevalent in the hot and arid region between the east of Asia to the northern part of Africa. The dromedary camel was first domesticated around five thousand or six thousand years ago in the Arabian region (Trinks et al., 2012; Almathen et al., 2016). Bactrian camels are found in cold regions and desert of central Asia. Small camelids include the llama and the alpaca which are restricted to South America. A less widely present and reported form of large camelids is the tartary camel (*C. bactrianus ferus*) which are found in the remote areas of Mongolia and China. These wild bactrian camels are considered the lone survivors of Old World Camels (Bannikov, 1976; Ji et al., 2009). Their total number reported in 2012 was 730–880. This species is facing a population size reduction of at least 80% within the next 45–50 years (Hare, 2014).

Camels have several unique traits which enable them to survive and live in remote areas, high mountains and arid lands. They can withstand thirst and hunger for long periods in the most inhospitable ecological conditions. Genetic studies of camel have elucidated the role of several genes that enable them to adapt to desert conditions. Importantly, several studies have uncovered the beneficial property of camel products in the treatment of various diseases (Muyldermans et al., 2009; Agrawal et al., 2011; Jirimutu et al., 2012; Fitak et al., 2016).

This review covers several features of the Camelidae family, which distinguishes them even from closely related species. Some of the unique biological attributes and genomic variations make them fascinating not only for monetary purposes but also for the treatment of human ailment.

## Camel Distribution

**FIGURE 2:**
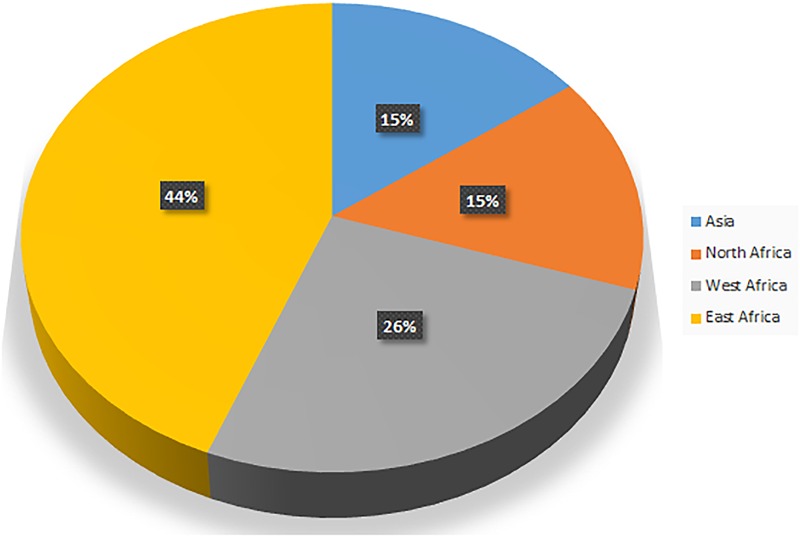
Camel distribution by region.

The camel number and distribution vary from region to region. While human population living in deserts has substantially decreased, the trend of nurturing camels in the desert is increasing at the global level. The camel population was estimated to be 27 million in 2014 according to the Food and Agriculture Organization (FAO). Interestingly, the annual growth rate of camel is higher than sheep, cattle, and horse (Faye and Bonnet, 2012). According to FAO statistics, the camel population is increasing with a yearly growth of 2.1%. The proportion of camels in the Domestic Herbivorous Biomass (DHB) was 1.1% in 1961 and this has increased to 1.5% in 2014 (Faye, 2016). Previously, camels were associated with nomadic communities. However, they are now an important part of modern life in arid regions (Breulmann et al., 2007). The distribution of camel population by region is given in Figure 2 (Faye, 2016). More than 80% of the world’s camel population is estimated to be in Africa. The camel population ranges from 120,000 to 998,000 in countries such as Turkmenistan, Egypt, Kazakhstan, Afghanistan, Tunisia, Oman, Saudi Arabia, Nigeria, China, Mongolia, Algeria, Eritrea, India, United Arab Emirates, Yemen, and Mali (Faye, 2016). Based on FAO statistics, the top 20 countries with camel population are listed in Table 1 (Food and Agriculture Organization [FAO], 2018). Faye and co-workers investigated the growth rate of camel in different countries and listed the countries with increasing and declining growth rates. They concluded that a decline in camel growth rate is due to a reduction in arid land devoted to camel rearing and a lack of universal selection programs for choosing the best breeds.

**Table 1:** Top 20 countries with camel population.

Sl. No	Country	Continent	Population (in millions)
(1)	Somalia	Africa	7.15
(2)	Sudan	Africa	4.79
(3)	Kenya	Africa	2.93
(4)	Niger	Africa	1.72
(5)	Chad	Africa	1.55
(6)	Mauritania	Africa	1.39
(7)	Ethiopia	Africa	1.16
(8)	Pakistan	Asia	1.02
(9)	Mali	Africa	0.97
(10)	Yemen	Asia	0.46
(11)	United Arab Emirates	Asia	0.40
(12)	India	Asia	0.38
(13)	Eritrea	Africa	0.37
(14)	Algeria	Africa	0.35
(15)	Mongolia	Asia	0.34
(16)	China	Asia	0.31
(17)	Nigeria	Africa	0.28
(18)	Saudi Arabia	Asia	0.27
(19)	Oman	Asia	0.25
(20)	Tunisia	Africa	0.24

*Total camel numbers are presented in million along with the continent.*

## Popular Camel Breeds

Several different breeds of camels exist and they are known locally under various names. Majority of camel breeds are differentiated based on phenotypic characteristics (Abdallah and Faye, 2012). The most common camel breeds are listed in Table 2.

**Table 2:** Popular camel breeds by country.

Sl. No.	Country	Breed	Coat color	Potential use	Reference
(1)	Algeria	Azawad	Light/white coat color	Racing camel	Aissa, 1989; Amine et al., 2013; Cherifi et al., 2017
(2)		Ouled Sidi Cheikh	Dark coat color	Dairy camel	
(3)		Rguibi	Clear/white coat color	Dairy camel	
(4)		Barbari	Various coat color	Dairy camel	
(5)		Regbi	Light color coat	Racing camel	
(6)		Targui	White/clear coat color	Racing camel	
(7)		Hamra	Reddish brown coat color	Multipurpose camel	
(8)	China	Alashan	Apricot yellow, purple, brown, and white color coat	Multipurpose camel	Ming et al., 2017
(9)		Qinghai	Sandy beige and puce coat color	Dairy camel	
(10)		Tarim	Brown and yellow coat color	Multipurpose camel	
(11)		Shunite	Apricot yellow and purple red coat color	Multipurpose camel	
(12)		Xinjiang	Brown and yellow coat color	Multipurpose camel	
(13)	Egypt	Falahi	Various coat color	Transportation and agricultural purpose	Mukasa-Mugerwa, 1981; Ramadan and Inoue-Murayama, 2017
(14)		Maghrabi	Various coat color	Dairy camel	
(15)		Mowalled	Various coat color	Multipurpose camel	
(16)		Somali	Off-white coat color	Racing camel	
(17)		Sudani	Various coat color	Racing camel	
(18)	India	Bikaneri	Light brown to dark brown and dark red to brown red coat color	Multipurpose camel	Mehta et al., 2006; Mehta, 2014
(19)		Jaisalmeri	Light brown	Racing camel	
(20)		Kachchhi	Brown to dark brown coat color	Dairy camel	
(21)		Mewari	Light brown to dark brown, and some have white coat color	Multipurpose camel	
(22)		Malvi	Off-white coat color	Multipurpose camel	
(23)	Mongolia	Hos Zogdort	Various coat color	Multipurpose camel	Chuluunbat et al., 2014
(24)		Galbiin Gobiin Ulaan	Various coat color	Multipurpose camel	
(25)		Galba Gobi	Red coat color	Dairy camel	
(26)		Khaniin Kheziin	Brown coat color	Dairy camel	
(27)	Pakistan	Brela	Blackish brown to light brown coat color	Dairy camel	Raziq et al., 2010, 2011
(28)		Kohi	White coat color	Multipurpose camel	
(29)		Marrecha	Blackish brown to light brown coat color	Multipurpose and racing camel	
(30)		Kutchi	Brown to dark brown coat color	Dairy and racing camel	
(31)		Pahwali	Dark brown to black	Multipurpose camel	
(32)		Peshin	Light brown to dark brown	Dairy camel	
(33)	Saudi Arabia	Aouadi	Red to white coat color	Multipurpose vocation camel	Abdallah and Faye, 2012; Massad, 2012; Abdelrahman et al., 2013; Al-Atiyat et al., 2016
(34)		Asail	Yellow to brown color	Racing camel	
(35)		Awrk	White coat color	Multipurpose vocation camel	
(36)		Hadhana	Light brown coat color	Multipurpose vocation camel	
(37)		Hamor	Brown coat color	Dairy camel	
(38)		Maghateer	White coat color	Multipurpose vocation camel	
(39)		Majaheem	Black coat color	Dairy camel	
(40)		Safrah	Dark brown coat color	Dairy camel	
(41)		Saheli	Red coat color	Multipurpose vocation camel	
(42)		Shaele	Gray coat color	Dairy camel	
(43)		Shageh	Gray coat color	Racing camel	
(44)		Sofor	Dark brown coat color	Dairy camel	
(45)		Waddah	White coat color	Dairy camel	
(46)		Zargeh	Blue-gray coat color	Racing camel	
(47)	Somalia	Eyddimo	White coat color	Multipurpose camel	Rao et al., 1970; Husein, 1987
(48)		Hoor	White coat color	Dairy camel	
(49)		Sifdar	Gray to reddish coat color	Multipurpose camel	
(50)	Sudan	Al Anafi	Yellowish white coat color	Racing camel	Hjort af Ornäs and Dahl, 1991; Wardeh, 2004; Ishag et al., 2010; Volpato, 2017; Ramadan and Inoue-Murayama, 2017
(51)		Al Bishari	White or yellowish coat color	Racing camel	
(52)		Al Arabi	Sandy gray, or fawn coat color	Dairy camel	
(53)		Ould Sidi Al Sheikh	Light coat color	Dairy camel	
(54)		Kabbashi	Red, gray, and yellow coat color	Dairy camel	
(55)		Kenani	Dark brown, gray, and yellowish coat color	Multipurpose camel	
(56)		Lahwee	Brown, red, and yellowish coat color	Multipurpose camel	
(57)		Piebald	White and solid (black, brown, tawny, red, or gray) coat color	Dairy and aesthetics purpose camel	
(58)		Rashaidi	Dark gray and pinkish red coat color	Dairy camel	
(59)		Shallageea	Various color coat	Dairy camel	

*Name of the breed and their identifiable coat color phenotype is also mentioned along with their most common use.*

Normally, each breed has a unique genetic makeup. However, traditional breeding is based on color phenotypes, which are correlated with certain economic and behavioral traits. With the increasing demand for sustainable food sources in desert regions, the interest in economic traits is also growing. Based on inherent local knowledge, economic traits (higher milk yield, drought resistance) are correlated with color phenotypes. With the advent of rapid genetic screening, beneficial traits in different breeds of camels are now being linked to mitochondrial and microsatellite markers.

It was observed that dromedary breeds of Nigerian origin are composed of a homogenous gene pool and no clear breed (Kurri and Kala) differentiation was noted by using molecular methods (Abdussamad et al., 2015). Ishag et al. (2010) studied single nucleotide polymorphism (SNP) in the growth hormone (GH) gene of six Sudanese camel breeds (Kenani, Lahwee, Rashaidi, Anafi, Bishari, and Kabbashi) using restriction fragment length polymorphism (RFLP). They identified a SNP 419C>T in intron 1 and determined that frequency of the C allele was high in pack camels (heavy weight) when compared to riding camel (light weight) (Bishari and Anafi). This substitution, associated with higher growth and body weight, has been used as a selective marker to assess genetic biodiversity in camel breeds (Abdel-Aziem et al., 2015). The association between GH polymorphism and body weight was also evaluated in four breeds of Arabian camels (Saheli, Majaheem, Waddah, and Homor) (Afifi et al., 2014). One SNP was identified in Waddah and Homor breeds at position 419C>T, two SNPs in Saheli breed at 419C>T and 450T>C, and thirteen SNPs in Majaheem breed. Together, these studies concluded that SNP 419C>T and 450T>C were correlated with greater body weight (Ishag et al., 2010; Afifi et al., 2014).

## Physiological Adaptation to Arid Conditions

The camel possesses inimitable characteristics which enable them to survive in extreme desert conditions. They store energy in their humps in the form of fat which enables them to survive for longer period without food and water (Emmanuel and Nahapetian, 1980). The body temperature of camels can fluctuate from 34 to 41°C within the day (Schmidt-Nielsen et al., 1956). It has been reported that camels can easily lose water that is equivalent to more than 25% of its body weight (Macfarlane et al., 1963). Another study concluded that camels could lose water up to 30% of its body weight during dehydration while other mammals could die due to circulatory failure when the water loss exceeds 12% of their body weight (McKinley et al., 2001). Water balance in the body is controlled by several factors including tissue osmolality and most importantly blood osmolality (Andersson et al., 1980). Except camels, all mammalian erythrocytes are concave or spherical in shape. However, in camels it is highly ovaloid, flat, small and enucleated and circulates in large numbers (Goniakowska-Witalinska and Witalinski, 1976; Azwai et al., 2007). The unusual elliptical shape of camel red blood cells facilitates their flow in a dehydrated state and makes it possible to cross small capillaries (Eitan et al., 1976). Additionally, camel erythrocytes are very resistant to osmotic hemolysis and are able to swell up to 240% of their original volume without bursting (Oyewale et al., 2011). This might be due to the altered distribution of membrane phospholipids in its red blood cells (Warda and Zeisig, 2000). Kidneys of camels play a major role in the process of conservation of water through increasing the osmolarity of urine. It has a strong water reabsorption capacity and eliminates highly concentrated urine. The intestine of camels also reabsorbs water and, unlike other mammals, water loss through feces is low since the feces is very dry (Davidson, 2014). Interestingly, camels deal with high blood glucose level (twofold than other ruminants) without developing diabetes. They can also consume eight times more salt than sheep and cattle without showing any signs or symptoms of hypertension (Al-Ali et al., 1988; Ali, 1994; Jirimutu et al., 2012).

## Camel Genome

While camels have enormous cultural, economic and biological importance, very little is known about the camel genome and the evolutionary advantages it imparts. Domestic animals and livestock, including camels, hold unique features and genetic variations (Andersson and Georges, 2004). The first draft of both domestic and wild bactrian camel genomes were reported in 2012. The size of bactrian camel genome was reported as 2.38 Gb and contained 20,821 genes (Jirimutu et al., 2012). This was close to the camel genome size (2.02–2.40 Gb) estimated based on haploid DNA content (*C*-value). Based on phylogenetic analysis, the authors also revealed that the camel shared a common ancestor with even-toed ungulates around 55–60 million years ago. Immunoglobulin-like domains were the most common proteins found in the camel genome and the results were consistent with a previous report. After annotation, results also suggested that the rhodopsin-like G protein-coupled receptor family, which is widely involved in regulating signaling pathways of physiological and biological processes, was the most abundant protein family found in the genome. The authors also searched for heavy-chain antibodies (HCAbs) in the bactrian camel genome that are homologous to the human, alpaca and dromedary IgH gene. 17 VH (heavy-chain variable region), 7 DH (diversity region), 6 JH (joining region), and 10 CH (constant region) genes were identified. Their results showed that VH genes of bactrian camel contains mutations in the coding region of HCAbs which prevent them from binding to the light chains. Further, they predicted the presence of 20,821 bactrian camel genes. Additionally, each gene contained an average of 8 exons and 1,322 bp coding regions (CDS). Importantly, GC content of the CDS region was estimated to be 52% which was significantly higher than the whole genome. Wernersson et al. (2005) also reported similar differences in other mammals. A similar report, related to sequencing the genome of bactrian, dromedary and alpaca camels, was published in 2014. The genome size of bactrian camel reported in this study (2.45 Gb) was similar to the earlier report (Wu et al., 2014). Additionally, the authors reported that non-coding RNA genes of dromedary, bactrian and alpaca genomes shared similar copy numbers. Analysis of the bactrian camel genome showed that 34% of the DNA is repetitive which is lower than human (>50%) and other mammals (Lander et al., 2001; Elsik et al., 2009; Wade et al., 2009; Wu et al., 2014). However, this is close to dogs (34%) and mice (35%) (Waterston et al., 2002; Lindblad-Toh et al., 2005). Most of the repetitive DNA is present in the form of transposon derived repeats in bactrian camels. It was observed that long scattered elements are similar to other mammals whereas short interspersed elements have a lower frequency in bactrian camels when compared to other mammals. Based on these results it was suggested that this might be one of the reasons why the camel has a smaller genome size than other mammals (Jirimutu et al., 2012). Interestingly, primate genomes have multiple copies of Alu repeats. However, none exists in the bactrian camel genome. Additionally, 244,141 microsatellites or simple repeats loci were found in the camel genome which could be helpful in marker assisted selection among camels and quantitative trait locus mapping. A comparison of the features of camel genomes and other species are presented in Tables 3, 4.

**Table 3:** Genomic comparison of bactrian camel and other mammals.

Species	Short interspersed elements (%)	Long interspersed elements (%)	Genome size (Gb)	Number of syntenic blocks
Bactrian camel	4	19	2.38	1,100
Cattle	18	23	2.9	1,121
Horse	7	20	2.7	1,005
Human	13	21	3.2	1,044
Mouse	8	19	2.5	1,070

**Table 4:** Genomic comparison of different camel species.

Genomic characteristic feature	Bactrian camel	Dromedary camel	Alpaca camel	Reference
Number of genes	20,251	20,714	20,864	Jirimutu et al., 2012; Wu et al., 2014; Khalkhali-Evrigh et al., 2018
GC content (%)	41.3	41.2	41.4	
Repeat content (%)	30.4	28.4	32.1	
Expanded genes	183	373	501	
Contracted genes	753	853	2189	
Non-coding RNA gene-copy number	1942	2209	2328	
Species specific homologous gene families	156	153	296	
Heterozygote rates	1.16 × 10^-3^	0.74 × 10^-3^	2.66 × 10^-3^	

Assessment of genetic variability among livestock species is critical to the regulation of genetic resources for their sustainable conservation and utilization. Characterization of genetic diversity within and among different breeds/populations is the prime strategy for management of animal biodiversity, mostly used in recognizing genetically unique structure (Excoffier and Lischer, 2010). Such steps are very important in the case of camels because their numbers have shown a rapid decline in the last decade. An innovative method of genome selection using dense marker maps covering all chromosomes suggested by Meuwissen and co-workers and the advent of next generation sequencing technologies have revolutionized the application of genetic information in livestock breeding programs (Hayes and Goddard, 2001). The proper implementation of genomic selection programs in different livestock species have produced remarkable results with regard to increasing the rate of genetic improvement (Hayes et al., 2009; Duchemin et al., 2012). Moreover, the estimation of large-scale genetic variation, particularly SNP markers, using whole genome sequencing could lead to the development of methods such as genome-wide association studies and genomic selection in camel breeding. Notwithstanding recent studies on camels at the genomic level, numerous genetic variants in different camel breeds remain to be identified and correctly annotated.

Heterozygosity is of significant interest in the study of genetic variations in populations. Jirimutu et al. (2012) conducted a study to determine heterozygous SNPs in wild and domestic camel genomes. The heterozygosity rate was calculated to be around 1.0 × 10^-3^ across the whole genome in both cases. Additionally, small indel number was also comparable between the two genomes. The authors further classified the SNPs in terms of gene annotations and measured the heterozygosity rates for coding and non-coding regions. Interestingly, domestic camel genome contained lower heterozygosity rate in the exonic region as compared to wild camel genome. This seems to support the idea of artificial selection of certain genes in the domestic species since strong artificial selection would reduce genetic diversity around specific loci (Doebley et al., 2006). The authors reported 2,816 regions containing 196 genes where the heterozygosity rate of the domestic camel was significantly lower than the wild one. Such genes were highly enriched in membrane receptors, particularly olfactory receptor activity, based on gene ontology analysis. Therefore, it was suggested that olfaction may be an important object of artificial selection during the domestication of bactrian camels. Further, Vijh et al. (2007) determined genetic variability and relationship among four camel breeds (Bikaneri, Jaisalmeri, Kutchi, and Mewari) using 23 microsatellite loci. A total of 252 alleles were detected across all the four populations. Interestingly, the calculated heterozygosity for the four breeds was 0.58 (Bikaneri), 0.57 (Jaisalmeri), 0.56 (Kutchi), and 0.60 (Mewari), which were lower than the expected heterozygosity value. Apart from this, mitochondrial DNA (mtDNA) has also been used to determine the genetic diversity and phylogeography in many animals (Chen et al., 2005; Lai et al., 2006). Researchers studied an 809 bp mtDNA fragment in 113 individuals from seven Chinese breeds, one Russian breed, three Mongolian breeds, two hybrid individuals and one wild breed to measure the genetic diversity and phylogeography of bactrian camel populations (Ming et al., 2017). They found 15 different haplotypes and the phylogenetic analysis proposed that domestic and wild bactrian camels belonged to two distinct lineages. Molecular variance analysis further suggested that wild and bactrian camels have different maternal origin. However, no significant genetic divergence was observed among domestic bactrian camels that belonged to different geographical locations. These findings implied that genetic admixture and gene flow might exist between these domesticated bactrian camels.

A recent study reported the first whole genome resequencing of individual camels from two distinct geographical regions (Khalkhali-Evrigh et al., 2018). In this study 692,908 indels and 4,727,238 SNPs were identified in the two camels. It was observed that most SNPs and indels were located in intergenic region based on *in silico* functional analysis. Additionally, the authors found 15,168 non-synonymous SNPs which were common to the three breeds (Yazd, Trod, and African dromedary) that could affect gene function and protein structure. In spite of this, much more needs to be done to improve our understanding of the camel genome and its role in breeding and genomic selection.

## Genetic Adaptation to Arid Conditions

Besides physiological studies, genomic and transcriptomic analyses have recently unraveled the peculiarities of the unusual adaptations in camels (Jirimutu et al., 2012; Wu et al., 2014). Studies have investigated the role of ‘rapidly evolving genes’ in species differentiation and adaptation in camels (Kasahara et al., 2007; Muyldermans et al., 2009; Jirimutu et al., 2012; Wang et al., 2012). Rapid divergence of protein-coding genes are normally calculated by an increased ratio of non-synonymous-to-synonymous substitutions (dN/dS) (Jirimutu et al., 2012). Jirimutu et al. (2012) identified around 2,730 significantly faster evolving genes in camels than its closest cattle orthologs. These genes were enriched in metabolic pathways such as carbohydrate and lipid metabolism, insulin signaling pathways and adipocytokine signaling pathways. They hypothesized that these genes might have helped the camel to optimize their energy storage and production in the desert. Generally, monogastric animals have high blood glucose levels (3.5–5.0 mmol/l) than ruminants (2.5–3.5 mmol/l) (Elmahdi et al., 1997). The camel is a ruminant herbivores with an extensive forestomach. However, it has a high blood glucose level (6–8 mmol/l) when compared to other mammals. The results suggest that rapidly evolving genes like CYP2E and CYP2J could be involved in type II diabetes mellitus (Jirimutu et al., 2012). Two critical genes in the insulin signaling pathways – PI3K and AKT – have undergone rapid divergence in camels which could have changed their response to insulin (Wang et al., 2012). This finding strongly supports previously reported physiological experiments that demonstrated that high glucose level in camel blood is due to their strong insulin resistance (Kaske et al., 2001). The distribution of cytochrome P450 (CYP) genes, which are involved in the arachidonic acid metabolism were found to be quite different in camels when compared to other mammals. Genome sequence analysis of bactrain camels found a higher number of copies of the cytochrome P450 (CYP) genes such as CYP2J (11 copies) and CYP2E (2 copies) in camels when compared to closely related mammals and humans. But CYP4A (one copy) and CYP4F (two copies) genes were fewer than other mammals (Jirimutu et al., 2012). CYP2E and CYP2J help to transform arachidonic acid into 19(S)-hydroxy-eicosatetraenoic acid [19(S)-HETE], whereas CYP4F and CYP4A transform it into 20-HETE (Wang et al., 2012). 19(S)-HETE is a potent vasodilator of renal preglomerular vessels that stimulate water reabsorption and is potentially useful for the survival in deserts (Carroll et al., 1996). In addition, they also reported that multiple copies of CYP2J genes give them the ability to take large amount of salt without developing hypertension (Wang et al., 2012). The activity of CYP2J2 is regulated by high-salt diet and the suppression of this gene can lead to high blood pressure (Zhao et al., 2003). A study on the effects of dehydration on camel plasma levels of sodium and water-retaining hormone systems including the renin-angiotensin system, aldosterone and antidiuretic hormone, dehydration identified significant increase in serum sodium, creatinine, urea and plasma arginine vasopressin (AVP) levels while plasma aldosterone level was altered very little (Ali et al., 2012). These results agree with earlier reports that suggested that renin-angiotensin system is important for the maintenance of water balance during dehydration. Studies have also reported an increase of serum urea and creatinine levels during dehydration (Siebert and Macfarlane, 1971; Finberg et al., 1978). Camels are also able to withstand starvation while maintaining a persistent nitrogen level through urea-nitrogen recycling (Mousa et al., 1983).

Proteomic studies of dromedary organs have also been undertaken to explain a number of cellular mysteries related to surviving in arid conditions. The overexpression of α-actinin in the heart suggested an ability to adapt to fluctuations in blood concentration associated with alternative drought-rehydration periods. An increased expression of H^+^-ATPase in the brain is believed to provide a rapidly usable supply of energy (Warda et al., 2014).

Guanidinoacetate methyltransferase in the liver has a regulatory effect on high energy phosphate. It is a key enzyme involved in creatine phosphate synthesis, which is an important energy currency of the cell and has a protective role on Na^+^ and K^+^-ATPase. The hump fat tissue and kidney protein expression have been shown to favor cellular acidosis (Warda et al., 2014). Hump fat contained more proteins, well developed cytoskeleton and high levels of vimentin. The role of vimentin in the signal transduction pathway from a specific adrenoreceptor, β3 adrenoceptor (β3AR), to the activation of extra cellular signal regulated kinase (ERK) and its role in lipolysis make them an early marker of adipogenesis (Kumar et al., 2007). Additionally, the high level of glucagon in camel, with subsequent elevated basal blood glucose, is consistent with the role of vimentin in glucose transporter induced glucose adipocyte transport (Abdel-Fattah et al., 1999; Guilherme et al., 2000). These results strongly support the modulatory role of adipocyte vimentin as a reason for the tolerance of high blood glucose level in camels. Vimentin might operate as an inducer of a cellular trap for glucose and the abundance of vimentin in camel adipocytes could aid the morphology of the hump of the well-nourished camel and could be an adaptation for survival in arid conditions (Warda et al., 2014).

Camel hemoglobin is an interesting case study of adaptation to extremely high temperatures (Oyewale et al., 2011). Camel blood has a high concentration of glucose. However, hemoglobin (Hb) exhibits low glycation and exhibits higher electrophoretic mobility than hemoglobin from cattle or humans (Bazzi et al., 2013). Glycated hemoglobin A1C (HbA1C) is normally associated with diabetes. Glycosylated hemoglobin is formed by non-enzymatic attachment of glucose to N-terminal valine and internal lysine amino groups of hemoglobin (Rohlfing et al., 2002; Soranzo, 2011). The reaction between Hb and glucose is slow and irreversible (Higgins, 2012). The process of glycosylation in eukaryotes is based on the interaction between amino acid and glucose and could be categorized into five types: O-linked, C-linked, N-linked, P-linked, and G-linked (Chauhan et al., 2013). There is a direct relationship between HbA1C and blood glucose level in human and most other animals (Nathan et al., 2008; Bazzi et al., 2013). However, camels behave differently in the presence of high blood glucose. An *in silico* analysis on hemoglobin characteristics reported that hemoglobin beta chain (HBB) is resistant to N- and O-linked glycosylation especially in camels whereas, hemoglobin alpha chain (HBA) is susceptible to O-linked glycosylation. It has been reported that these factors, together with other post-translational modification, might be responsible for the protection of Hb from glycosylation (Thanka Christlet and Veluraja, 2001; Borai et al., 2011).

The unusual genetic architecture of camel is the reason behind its survival in harsh environmental conditions. The regulation of different genes in response to different stresses are summarized in Table 5.

**Table 5:** Regulation of camel genes under stress.

Camel physiology	Gene/Protein	Function/protein	Action	Reference
Oxidative stress	Nuclear factor, erythroid 2 like 2 (NFE2L2)	Antioxidant	Up-regulated	Huang et al., 2001; Barnes et al., 2002; Yancey, 2005; Burg et al., 2007; Gallazzini and Burg, 2009; Cheung and Ko, 2013; Wu et al., 2014
	Microsomal glutathione S-transferase 2 (MGST2)	Antioxidant	Up-regulated	
Water restricted conditions	Epithelial sodium channel (ENaC)	Controls the reabsorption of sodium in kidney	Up-regulated	
	Sodium/potassium transporting ATPase (Na^+^/K^+^-ATPase)	Sodium/potassium transporting ATPase	Up-regulated	
	Aquaporin 1 (AQP1-3)	Water channel protein permits passive transport of water in kidneys	Up-regulated	
	Nuclear factor of activated T-cells 5 (NFAT5)	Regulates gene expressions induced by osmotic stress	Down- regulated	
	Solute carrier family 5 member 3 (SLC5A3) also known as (SMIT)	Exhibit transporter activity and myo-inositol: sodium symporter activity	Down-regulated	
	Solute carrier family 6 member 6 (SLC6A6)	Regulates a family of sodium and chloride-ion dependent transporters	Down- regulated	
	Betaine/GABA transporter-1 (BGT1)	Regulates sodium symporter activity and gamma-aminobutyric acid	Down-regulated	
	Glucose transporter 1 (GLUT1)	Transport glucose into cells from blood	Up-regulated	
	Aldose reductase (AR)	Catalyzes reduction of glucose to sorbitol	Up-regulated	
	Sorbitol dehydrogenase (SDH)	Accumulation of sorbitol serves as a source of energy in WR	Down-regulated	
Heart physiology	Alpha actinin 2	Coupling of Ca^2+^ -activated K^+^ channel to L type Ca^2+^ channel	Up-regulated	Schmidt-Nielsen et al., 1981; Hoover et al., 2000; Lu et al., 2009; Warda et al., 2014
	Alpha B-crystallin	Small heat shock protein. Cytoprotective	Up-regulated	
	ATP synthase beta subunit	Proton leakage in cardiac muscles	Down-regulated	
	Isocitrate dehydrogenase		Up-regulated	
Liver physiology	Guanidinoacetate methyltransferase	Creatine phosphate synthesis and Antioxidant	Up-regulated	Kolling and Wyse, 2010; Wang et al., 2012; Warda et al., 2014
	14-3-3protein epsilon (14-3-3E) (Mitochondrial import stimulation factor L subunit) (MSF L) isoform 1	Control protein kinases and other cellular events including autophagy and tumorigenesis	Up-regulated	
	Glutathione peroxidase	Antioxidant and prevents heat-induced and acid-induced amorphous aggregation of proteins	Up-regulated	
Hump fat physiology	Cytochrome B	Electron carrier	Up-regulated	Dettin et al., 2003; Warda et al., 2014
	Galectin-1	Control basic cellular process	Up-regulated	
	β-galactoside-binding soluble 1 (L-14-I)		Up-regulated	
Brain physiology	β-Synuclein	Prevent neurodegeneration and inhibiting the fibrillation of α-synuclein	Up-regulated	Yamin et al., 2005
Thermal and dehydration stress	Heat shock protein 27, 65, and 73	Provide defense against dehydration or thermal stress in arid environments.	Up-regulated	Ulmasov et al., 1993; Warda et al., 2014

## Role of Camel Genome in Economics, Dairy and Sports

Pastoralist survival in the desert relies heavily on camels. Recent advancement in genetic engineering, mainly in livestock and the incorporation of concepts of selective breeding with desired phenotypes, has made camel rearing a profitable profession in numerous countries (Beuzen et al., 2000; Olesen et al., 2000). SNPs in the camel genome play a vital role in the development of useful traits. Such genetic variations are listed in Table 6.

**Table 6:** Single nucleotide polymorphisms (SNPs) linked with dairy potential in camels.

Gene name	SNP position	Potential influence	Reference
β-casein (CSN2)	2126A>G 6040G>A	Associated with milk production and composition traits	Pauciullo et al., 2014
αs1-casein (CSN1S1)	942G>T		Giambra et al., 2013
Growth hormone (GH) gene	419C>T450T>C	Increase body weightIncrease body weight	Ishag et al., 2010
Myogenic factors 5 gene (MYF5)	377A>T	Thickness of longissimus dorsi muscle	Af El-Kholy et al., 2016

Camel sport is an integral part of communities in the Middle East region. Many individuals adopt rearing of racing camels as a profession and regular events are organized. The demand for special breeds of racing camels, with higher endurance and resilience, is quite high. Developing novel techniques to measure oxidative stress and endurance in racing camels has very high potential (Wilson, 1999). Several regions have declared camel as their national animal and organizes events at the national level to promote and highlight camel rearing among local residents.

The mitochondrial DNA (mtDNA) of dromedary camel is 16643 bp in size and encodes 23 subunits of the electron transfer chain associated with the production of cellular adenosine triphosphate (ATP) through oxidative phosphorylation (Cui et al., 2007). The cells that require a lot of energy in terms of ATP, such as neurons and muscles, maintain high copy number of mtDNA (Dickinson et al., 2013). Polyacrylamide gel electrophoresis was used to analyze malate dehydrogenase (Mdh) and malic (ME) isoenzymes in Arabian camel for energy production and racing. Results indicated the necessity of the mitochondrial Mdh-2 for energy production in racing breed and cytosolic Mdh-1 for lipogenesis and energy production in both breeds (Al-Harbi and Amer, 2012). Likewise, the ratio of mitochondrial DNA (mtDNA) to nuclear DNA (nDNA) is also considered as a good predictor of the metabolic status of the tissue. This ratio was found to be high in racing camels when compared to dairy camels (Soman and Tinson, 2016).

## Camel Immunogenetics

Camels are relatively more resistant to major infectious diseases when compared to other livestock inhabiting the same geographical area. However, its immunogenome has not been well studied so far (Wernery and Kaaden, 2004; Muyldermans et al., 2009; Burger, 2016). The immune system of camels includes a unique heavy chain antibody homodimer. Camels are the only mammals that can produce heavy-chain antibodies (HCAbs) that lack the light chain (Hamers-Casterman et al., 1993; Tillib et al., 2014). However, little is known about the major histocompatibility complex (MHC) region of the camel genome (Antczak, 2013). The genomic MHC region contains immune response (IR) genes that play a crucial role in host–pathogen interactions (Janeway et al., 2001). Plasil and co-workers identified, mapped and characterized the MHC region of Old World camels.

The study investigated genomic localization, organization and sequence similarity of MHC genes and showed that camels possess MHC genes comparable to other mammals. Their results suggest that molecular diversity of MHC class II genes are significantly lower in Old World camels than in other mammals and the major part of the diversity resides in the DQB gene, which is not very well annotated in the camel genome (Plasil et al., 2016). MHC class I and class II genes contain antigen presenting molecules which are responsible for recognition of antigenic peptides expressed on the cell surface (Janeway et al., 2001). Other IR genes such as T cell receptors have evolved in camels due to mutations in the gamma (TRG) and delta (TRD) genes. Their genomic structure and organization have also been investigated (Ciccarese et al., 2014; Burger, 2016). New technology based on next generation sequencing could be used to probe and analyze the adaptive immune system. Millions of immunoglobulin sequences and T cell receptors from a single sample could be analyzed by amplifying in a single multiplex PCR reaction (Li et al., 2015). A study, which evaluated the sequence diversity of the constant and variable regions present in the 15 conventional heavy, 18 kappa and 35 lambda light chains of *C. dromedarius* and *C. bactrianus* by using standard Sanger sequencing, found that the functional sequences for these chains have been rearranged (Griffin et al., 2014).

## Camel Genome and Its Medicinal Potential

The unique features of the camel genome and proteome not only enable them to survive and thrive under harsh environmental conditions but also make them less susceptible to various pathological conditions. Camels employ several intrinsic immunological and molecular mechanisms against pathogenic agents and pathological conditions (Fazil and Hofmann, 1981).

### Camel Antibodies

**FIGURE 3:**
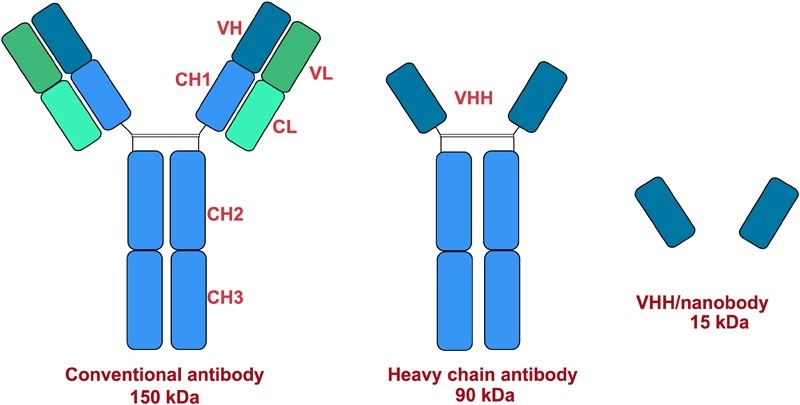
Structural models of antibodies.

Therapeutic antibodies are an important treatment option against several auto-immune, infectious and malignant diseases. Monoclonal antibodies (mAb) are preferred in targeted drug delivery because they are relatively cheaper and have high specificity, stability and potency (Finlay and Almagro, 2012). Three types of immunoglobulins (IgA, IgG, and IgM) were discovered and separated in camels (Grover et al., 1983; Azwai et al., 1993). Conventional γ-immunoglobulins consist of two heavy chains, each containing three invariant (constant) domains (CH1–CH3), a single variable region (VH), and two light chains composed of a constant region (CL) and a variable region (VL). The VH and VL regions together form the paratope and it is the antigen-recognizing portion of the antibody (Jerne, 1955). Interestingly, camel antibodies are different from other animals. In addition to its conventional heterotetrameric structure (two heavy and two light chains), camels also possess non-conventional antibodies which are smaller (Hamers-Casterman et al., 1993). These immunoglobulins are now known as heavy-chain antibodies (HCAbs). Non-conventional 2-chain IgG structures are devoid of the light chain and heavy chain constant region CH1 (Harmsen and De Haard, 2007). Structural representations of the different antibodies are shown in Figure 3. The antigen binding site of HCAb is a single variable domain denoted as VHH (variable domain of camel heavy-chain-only antibody) (Muyldermans, 2001; De Genst et al., 2006; Deschacht et al., 2010). VHHs have the ability to penetrate and target small antigenic sites that conventional antibodies are unable to recognize (Paalanen et al., 2011). Moreover, HCAbs are usually absent in other mammals except some primitive fish (Nuttall et al., 2001). The new antigen receptor (NAR) and the Cos5 antibodies are some examples of antibodies obtained from sharks (Greenberg et al., 1995). Camels and some primitive fish possess natural antibodies composed only of heavy chains. The antigen binding site is formed by only one single domain, referred to as VHH (Zielonka et al., 2014). Antibody derived VHHs have gained importance because of its low size (15 kDa), stability, binding abilities and enhanced potency (Vincke et al., 2012). Additionally, the production of such antibodies in camels are easier than other animals (Alvarez-Rueda et al., 2007). The camel germline IGHV, IGLV, and IGKV genes show high degree of sequence homology with human counterparts (Achour et al., 2008; Klarenbeek et al., 2015). Therefore, active immunization of camels with human target proteins results in a better diversity of potential therapeutic antibodies due to the presence of more foreign epitopes (Tainer et al., 1985). This degree of foreignness is best exploited by immunizing camels against viral envelope proteins which results in a diverse panel of binders, allowing targeting of multiple epitopes on the same envelope protein (Hultberg et al., 2011; McCoy et al., 2012). The potential uses of camel derived antibodies are mentioned in Table 7.

**Table 7:** Medical uses of VHHs derived camel antibodies.

Receptor/Protein/Antigen	Action	Medical use	Reference
Protein kinase C	Increases or decreases the activity	Type II diabetes mellitus	Paalanen et al., 2011
Lysozyme aggregation	Inhibitory	Parkinson’s and Alzheimer’s disease	Dumoulin et al., 2003; Chan et al., 2008
Poliovirus type 1 Sabin strain particles	Inhibit viral replication	Antiviral	Thys et al., 2010
Tumor associated antigen (TAA)	Anti-idiotypic vaccines	High risk cancer patients	Muyldermans and Lauwereys, 1999
Anti-complementary activity	Increase the activity which leads to decrease adverse reactions	Snake or insect antiserum	Meddeb-Mouelhi et al., 2003; Herrera et al., 2005
Tumor specific antigen	Senses or induces conformational changes	Prostate cancer	Saerens et al., 2004
Tumor specific antigen	Tumor immunolabelling	Diagnosis of tumors	Cortez-Retamozo et al., 2002
Dipeptidyl peptidase like 6 protein	Nanobody based tracer targeting DPP6	Imaging of human pancreatic endocrine cells	Balhuizen et al., 2017
Macrophage mannose receptor	Nanobody based tracer targeting macrophage mannose receptor	Imaging of joint inflammation	Put et al., 2013
Vascular cell adhesion molecule 1 (VCAM1)	Nanobody based targeting VCAM1	Imaging of atherosclerotic lesions	Broisat et al., 2012
Human epidermal growth factor receptor 2 (HER2)	Nanobody based targeting HER2	Imaging of HER2 receptors expression in cancer	Vaneycken et al., 2011; Xavier et al., 2013
CXCR4 nanobodies	Anti-proliferation, anti-metastatic and anti-angiogenic	Anti-HIV-1 entry activity	Jahnichen et al., 2010
CXCR7 nanobodies	Anti-proliferation, anti-metastatic and anti-angiogenic	Head and neck cancer	Maussang et al., 2013
Tumor necrosis factor alpha	Inhibitory	Rheumatoid arthritis (RA)	Coppieters et al., 2006
FcgammaRIII killer cells	Increases their recruitment	Detection and destruction of tumor cells	Behar et al., 2007
IL-6 receptor	Inhibitory	Rheumatoid arthritis	Mima and Nishimoto, 2009
Hepatocyte growth receptor	Nanobody based targeting hepatocyte growth receptor	Anticancer	Vosjan et al., 2012
Epidermal growth factor receptor (EGFR)	Nanobody based targeting EGFR	Anticancer	Roovers et al., 2011
vascular endothelial growth factor receptor-2 (VEGFR2)	Nanobody based targeting VEGFR2	Anticancer	Behdani et al., 2012
Carcinoembryonic antigen (CEA)	Anti-CEA nanobody CEA5	*In vivo* imaging of colon cancer	Leung, 2012
Capsid Protein L1	Nanobody based targeting major capsid protein L1	Cervical cancer	Minaeian et al., 2012
Death receptor 5 (DR5)	DR5 nanobodies lead to caspase activation	Antitumor	Huet et al., 2014

Several studies have explored the structure and discovery of nanobodies, as well as their applications in immunity, oncology, neurodegenerative diseases, infections, and other ailments (Wesolowski et al., 2009; Altintas et al., 2012; Steeland et al., 2016; Beghein and Gettemans, 2017). VHHs are highly suitable for oral immunotherapy due to their resistance to extremes of pH and their ability to bind to the target at high concentrations of chaotropic agents (Harmsen and De Haard, 2007). Furthermore, due to their binding specificities, they are considered excellent modulators or inhibitors of receptors, enzymes or viruses (Desmyter et al., 2013; Hassaine et al., 2014). Camel nanobodies exhibit properties that are very useful in crystallizing mobile proteins (Ward et al., 2013). It can also be used to stabilize membrane protein structures. Nanobodies with high isoelectric point (pI) have the potential to cross the blood brain barrier (BBB) and, in future, could be adapted for brain imaging purposes (Li et al., 2012). The current limitation is that a large amount of nanobody needs to be injected to achieve the desirable effect. G protein-coupled receptors (GPCRs) are normally stabilized in the presence of G protein. Current crystal structures provide insights into the inactive states of several GPCRs. It is difficult to obtain an agonist-bound active-state GPCR structure due to the inherent instability of this state in the absence of a G protein. Nanobodies have the potential to stabilize GPCR in the absence of G protein (Steyaert and Kobilka, 2011). Studies have employed nanobodies as a stabilizing agent to achieve the stabilization of agonist-bound form of the beta-2 adrenoreceptor (b2Ar) and human M2 muscarinic acetylcholine receptor (Rasmussen et al., 2011; Kruse et al., 2013). Comparison with the inactive structure reveals subtle changes in the binding pocket. Therefore, such structures provide insights into the process of agonist binding and activation.

### Camel Lactoferrin’s Role in Hepatitis C Virus

Hepatitis C virus (HCV) is a major health concern and is the main cause of chronic liver disease (Williams, 2006). It is estimated that over 180 million people suffer from this disease worldwide (Nguyen and Keeffe, 2005). The prevalence of HCV is likely to increase in the near future (Deuffic-Burban et al., 2007). Since no protective vaccine is available, the old treatment regimen of interferon alpha alone or in combination with ribavirin is currently widely adopted.

Lactoferrin (Lf) is a multifunctional glycoprotein that is usually present in milk. Its molecular weight is 80 kDa and it belongs to the transferrin family (Legrand et al., 2008). Lf also has a role as part of the innate immune system. Additionally, its antibacterial and antiviral potential have also been reported (Legrand et al., 2005). Lf has shown promising results against herpes simplex virus 1 and 2 (HSV-1 and HSV-2) (Marchetti et al., 1998). Lf also exhibits antiviral activity against HCV. The binding of human and bovine lactoferrin with envelope proteins of HCV inhibits the interaction of HCV virus with cellular receptors (Yi et al., 1997; Ikeda et al., 1998). These studies have shown that Lf shows its antiviral activity in the early stage of infection. Camel lactoferrin (cLf) inhibited the entry of HCV when cLf and HCV were preincubated (Redwan el and Tabll, 2007). More specifically, the C- and N-lobes of camel lactoferrin inhibits the activity of HCV (Redwan et al., 2014). Heparan sulfate (HS) is present on the cell surface of many animals. A host’s HS serves as a receptor for several pathogens. HS aids antiviral activity and binding of lactoferrin (Jenssen et al., 2004; Marchetti et al., 2004). It was demonstrated that camel lactoferrin has a higher antiviral activity against HCV than human, sheep and bovine lactoferrin (El-Fakharany et al., 2013). Apart from lactoferrin, camel milk casein has also shown promising activity against HCV. Furthermore, it has also been shown to possess apoptotic potential (Almahdy et al., 2011).

### Camel Urine as Anti-cancer Agent

**FIGURE 4:**
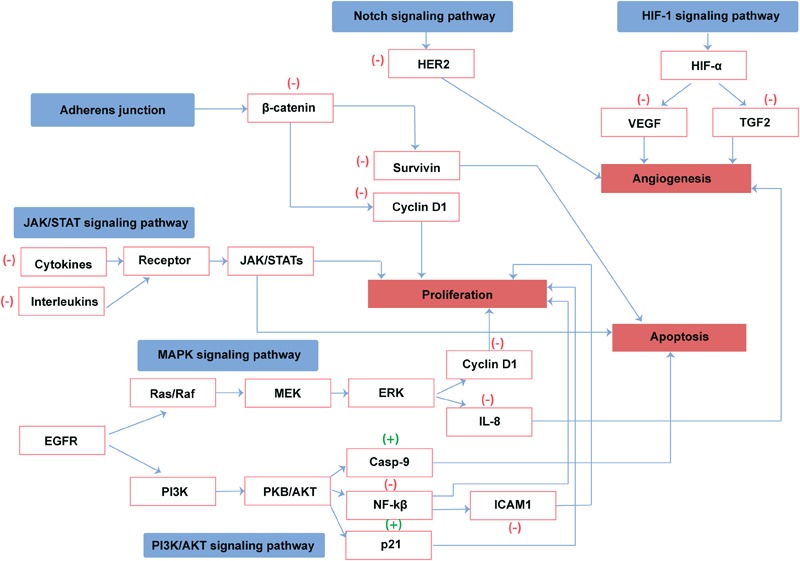
Reported direct and indirect effect of camel urine on proteins involved in cancer pathways. (+) represents activation and (–) represents the inhibition of the protein by camel urine.

Cancer is a leading health problem worldwide. Chemotherapy is still considered as a fundamental treatment modality irrespective of its toxic side effects and high morbidity (Rood et al., 2004). Most of the drugs used for the management of cancers are derived from plant sources (Craig, 1997). Camel urine is a natural product used for the management of several diseases in the Arabian region. Cancer patients usually drink (100 ml/day) camel urine alone or mixed with milk. Camel urine is devoid of bad odor and toxicity due to low urea and lack of ammonia. Additionally, camel urine is basic (pH > 7.8), while human urine could be weakly acidic or weakly basic (Read, 1925). Research on camel urine has shown that it has antifungal and antibacterial activity and is able to protect the liver from CCL4 induced damage (Al-Bashan, 2011; Alzahrani and Alharbi, 2011). Gastroprotective and ulcer healing effects of camel urine have also been reported (Hu et al., 2017). Camel urine has potential activity as antiplatelet and anticancer agents as well (Alhaidar et al., 2011). Camel urine inhibits the induction of CYP1A1 gene expression by 2,3,7,8-tetrachlorodibenzo-*p*-dioxin (TCDD). TCDD is a potent CYP1A1 inducer and a well-known carcinogen. This depicts the transcriptional regulation in which the binding of TCDD to a cytosolic transcription factor, the aryl hydrocarbon receptor (AhR), is the first step in a series of cellular events leading to carcinogenesis and mutagenesis. Research on camel urine has shown that camel urine, but not bovine, inhibits TCDD-mediated toxic effect by inhibiting the expression of CYP1A1 at both transcriptional and post-transcriptional levels (Alhaider et al., 2011). Good anticancer agents activate cell death or inhibit proliferation of tumor cells without affecting the growth of normal cells. Fortuitously, camel urine presents all these features (Al-Yousef et al., 2012). Anticancer activity of camel urine has also been demonstrated using GC-MS and ICP-MS methods (Ahamad et al., 2017). Studies using GC-MS and ICP-MS have shown a marked difference in urinary metabolites produced by camels. Camel urine metabolites like canavanine are also excreted by other mammals but the quantity is low when compared to camel. Canavanine, an arginine analog, is a by-product of amino acids and urea metabolism and it has been shown to possess potent activity against tumor cells. Camel urine has also displayed antimetastatic effect on breast cancer cells (Romli et al., 2017). Reported pharmacological activity of camel urine on different genes/proteins are listed in Table 8. A schematic diagram providing an overview of proteins and pathways affected by camel urine is given in Figure 4.

**Table 8:** Action of camel urine on proteins.

		Effect of	
Protein	Function	camel urine	Reference
Cyclin D1	Prooncogene	Inhibitory effect	Yang et al., 2006; Al-Yousef et al., 2012
Beta-catenin	Transcription factor	Inhibitory effect	Prasad et al., 2007; Al-Yousef et al., 2012
Bcl-2	Antiapoptotic protein	Inhibitory effect	Callagy et al., 2006; Al-Yousef et al., 2012
Survivin	Antiapoptotic protein	Inhibitory effect	Tanaka et al., 2000; Al-Yousef et al., 2012
Cyclin- dependent kinase inhibitor p21	Antiproliferative activity	Excitatory effect	Lacroix et al., 2006; Al-Yousef et al., 2012
Th2 cytokines Interleukin-4, Interleukin-10	Immunosuppressive and tumor stimulating growth factors	Inhibitory effect	Nagai and Toi, 2000; Mocellin et al., 2005; Romli et al., 2017
Interleukin-6	Potent growth factor cytokine	Inhibitory effect	Romli et al., 2017
Interleukin-1β	Proinflammatory cytokine	Inhibitory effect	Alhaider et al., 2014; Romli et al., 2017
Interleukin-4	Proinflammatory cytokine	Inhibitory effect	Alhaider et al., 2014; Romli et al., 2017
Transforming growth factor-β (TGF- β)	Proangiogenic and proinflammatory cytokine	Inhibitory effect	Wendt et al., 2012; Alhaider et al., 2014
Tumor necrosis factor-α (TNF- α)	Proangiogenic and proinflammatory cytokine	Inhibitory effect	Szlosarek and Balkwill, 2003; Alhaider et al., 2014
The chemokine (C-C motif) ligand 2 (CCL2)	Proangiogenic and proinflammatory cytokine	Inhibitory effect	Qian et al., 2011; Alhaider et al., 2014
vascular endothelial growth factor (VEGF)	Proangiogenic mediator	Inhibitory effect	Torimura et al., 1998; Alhaider et al., 2014
Caspase 3	Proapoptotic protein	Excitatory effect	Al-Yousef et al., 2012; Alhaider et al., 2014
Bax	Proapoptotic protein	Excitatory effect	Al-Yousef et al., 2012
CD3+/CD4+ T helper cells	Regulate immune response	Excitatory effect	Romli et al., 2017
CD3+/CD8+ cytolytic T cells	Regulate immune response	Excitatory effect	Romli et al., 2017
Nuclear factor (NF-κβ)	Stimulates proliferation, proinflammatory and antiapoptotic factor	Inhibitory effect	Gerondakis et al., 2006; Romli et al., 2017
Granulocyte-macrophage colony-stimulating factor (GM-CSF)	Proangiogenic cytokine	Inhibitory effect	Gutschalk et al., 2013; Romli et al., 2017
Intercellular adhesion molecule 1 (ICAM1)	Proinflammatory and prometastatic protein	Inhibitory effect	Maruo et al., 2002; Romli et al., 2017
Quinone oxidoreductase 1	Cancer protective gene	Excitatory effect	Korashy et al., 2012
Leptin	Involved in proliferation and metastasis	Inhibitory effect	Paik et al., 2009; Romli et al., 2017
Cytochrome P450 1a1	Prooncogene	Inhibitory effect	Alhaider et al., 2011; Korashy et al., 2012

### Camel Milk and Diabetes Mellitus

Diabetes mellitus (DM) is a group of metabolic disorders characterized by an elevated level of blood glucose. It results from the inability of body to produce enough insulin (type 1 diabetes) or the failure to respond efficiently to the insulin produced by the pancreatic beta cells (type 2 diabetes) (Diamond, 2003). Worldwide prevalence of DM is expected to be 4.4% by 2030 (Wild et al., 2004). Metabolic control of hyperglycemia can be achieved through physical activity, diet with or without antidiabetic agents, which will help to prevent the likely risk of chronic complications (Lindstrom et al., 2006). Camel milk has exceptional biochemical properties (Wernery et al., 2006). The unique constituents of camel milk and their stability at a range of physiological conditions are different from other mammals (Shamsia, 2009). Camel milk has been used for a long time for a number of ailments (Yagil, 1982). One of its major roles is in controlling hyperglycemia in type 1 diabetes (Agrawal et al., 2005; Khan et al., 2013). Camel milk does not form coagulum in acidic environment and has a high buffering capacity. However, milk of other mammals readily forms coagulum in acid milieu (Wangoh, 1993). It was observed that camel milk protein has insulin-like properties and the lack of coagulum formation in the stomach allows it to pass rapidly and remains available for absorption in the intestine (Beg et al., 1986). Clinical studies showed that administration of camel milk as adjunct therapy with insulin to patients of type 1 diabetes reduced insulin requirement by up to 30% (Agrawal et al., 2007). Camel milk contains insulin like proteins which potentiates its interaction with insulin receptors (Mehaia et al., 1995; Malik et al., 2012). Studies have been conducted to determine the cellular and molecular mechanisms behind the insulin like properties of camel milk and its ability to control hyperglycemia (Agrawal et al., 2011). However, the mechanism has not been clearly elucidated yet. By using bioluminescence resonance energy transfer (BRET), it was observed that camel milk activated the human insulin receptor (hIR). Though it had no direct insulin like effect, it significantly potentiated the effect of insulin when pre-treated with camel milk (Abdulrahman et al., 2016). Camel milk also has a different content of casein, higher amount of vitamin B3 and lipid micelles that could enhance the body’s defense against free radicals (Al-Humaid et al., 2010).

The complications of DM, like neurological and vascular alterations, may worsen in the presence of high oxidative stress (Varvarovska et al., 2004). Normally, antioxidant enzymes such as catalases, superoxide dismutase (SOD), and glutathione peroxidase are responsible for scavenging reactive oxygen species (West, 2000). Camel milk has also shown antioxidant activity most probably by its chelating effects on toxicants (Shori, 2015). Another complication of DM is delayed wound healing. The presence of infectious agents prevents normal healing of wound. Camel milk contains whey protein and lacks beta-lactoglobulin. It exhibits a higher antioxidant activity than bovine milk and other whey proteins due to a high content of antioxidant amino acids (Cys, Met, Trp, Tyr, and Phe) (Marshall, 2004; Salami et al., 2010). Therefore, camel whey protein speeds up healing by increasing the immune response of wounded tissue cells (Badr, 2013). Camel milk also comprises of different proteins such as serum albumin, lactophorin, peptidoglycan recognition protein, and alpha lactalbumin protein (Kappeler et al., 2004). These proteins bind to lactic acid bacteria and other gram-positive bacteria with an affinity similar to that reported for the human and murine orthologs.

## Conclusion

The camel has a special status among mammals domesticated by humans since they are highly adapted to the extreme desert ecosystem. It is a multipurpose animal used for dairy production, racing and transportation. The genetic makeup and variations in its genome allow them to survive these harsh conditions. The involvement and identification of key genes in the adaptation to desert environment may have applications in breeding programs. It may also provide insights into disease resistance in the future. Knowledge of the genetic differences between different types of camels may also assist in screening them for specific purposes like sports or dairy. Elucidation of the therapeutic role of camel products is ongoing. Clearly, further studies are required to decipher the precise molecular mechanisms that underpin their role in disease pathogenesis and the ability to stimulate the immune system.

## Author Contributions

All authors listed have made a substantial, direct and intellectual contribution to the work, and approved it for publication.

## Conflict of Interest Statement

The authors declare that the research was conducted in the absence of any commercial or financial relationships that could be construed as a potential conflict of interest.
